# Perceptions, attitudes, and practices of a Belgian teaching hospital's physicians, pharmacists, and nurses regarding antibiotic use and resistance: survey towards targeted actions for Antimicrobial Stewardship

**DOI:** 10.1186/s13756-023-01228-w

**Published:** 2023-03-19

**Authors:** Caroline Briquet, Youssra Khaouch, Jean Cyr Yombi

**Affiliations:** 1 Antimicrobial Stewardship, Cliniques Universitaires Saint-Luc, UCLouvain, 10 Avenue Hippocrate, 1200 Brussels, Belgium; 2 Department of Pharmacy, Cliniques Universitaires Saint-Luc, UCLouvain, 10 Avenue Hippocrate, 1200 Brussels, Belgium; 3 Department of Clinical Biology, Cliniques Universitaires Saint-Luc, UCLouvain, 10 Avenue Hippocrate, 1200 Brussels, Belgium; 4 Department of Internal Medicine and Infectious Diseases, Cliniques Universitaires Saint-Luc, UCLouvain, 10 Avenue Hippocrates, 1200 Brussels, Belgium

**Keywords:** Antimicrobial resistance, Antibiotic use, Antimicrobial Stewardship, Health professionals, Public health, COM-B model

## Abstract

**Objectives:**

This study aimed to identify barriers to the proper use of antibiotics by healthcare professionals and to help the hospital Antimicrobial Stewardship develop suitable actions for the staff.

**Methods:**

In a Belgian teaching hospital, a survey was conducted among physicians, pharmacists, and nurses involved in antibiotherapy. Questions from the 2019 European Center for Disease Prevention and Control (ECDC) survey were analyzed based on components of the COM-B model (*capabilities*, *opportunities*, and *motivations*). First, collected data were reviewed with the Ethnos software to analyze the different COM-B model components. For statistical analyses, responses were grouped into three clear-cut answers in a Fisher’s exact test.

**Results:**

Overall, 400 staff members were included. We found that our professions, combined, have a good perception of antibiotic resistance (97.8%). For capabilities, however, only 77.2% state that they have sufficient knowledge, with 91.3%, 71.5%, and 63.0% for physicians, nurses, and pharmacists, respectively. For opportunities (access to resources, information, and training), it is observed that 72.2% report having easy access to the guidelines they need to manage infections. In comparison, for 64.2% of the respondents, this information changed their opinion on the useless or inappropriate prescription, administration, and delivery of antibiotics. For 55.0%, this information has enabled them to change their practices. Finally, for motivations, 92.8% of respondents state that they know about the link between their practices and the emergence and spread of antibiotic resistance. However, only 65.0% of participants say they have a role in managing antibiotic resistance. We found that 5 out of 8 questions are significantly dependent on the profession: 2 inquiries related to capability, 1 to opportunity, and 2 to motivation.

**Conclusion:**

We found that responses to the ECDC questionnaire are related to the profession. While some topics are universal/cross-functional, others must be explicitly tailored to each professional category. Information is useless if not accessible. Communication and provision of documents are thus paramount.

**Supplementary Information:**

The online version contains supplementary material available at 10.1186/s13756-023-01228-w.

## Background

The emergence and spread of antibiotic-resistant bacteria threaten public health [1]. The results of a survey conducted by the European Center for Disease Prevention and Control (ECDC) in 2017 reveal that Belgium is one of the biggest prescribers of antibiotics. The same report shows that 28.1% of hospitalized patients had received at least one antibiotic on the day of the prevalence survey [2, 3]. To control antibiotic use within hospitals, the Belgian Antibiotic Policy Coordination Commission (BAPCOC) has implemented a series of actions monitored and reviewed by local Antimicrobial Stewardship (AMS) [4]. AMS was made mandatory in 2008 in all acute and chronic care facilities with at least 150 specialized geriatric beds [5, 6]. They are in charge, among other things, of training healthcare professionals and implementing necessary tools for adequately using antibiotics.

Several articles show that health professionals are generally aware of the seriousness of the antibiotic resistance problem but also reveal discrepancies between recommendations for the proper use of antibiotics and their daily practices [7–9]. The reasons put forward vary according to the professional category observed. Indeed, physicians’ prescribing decisions will be influenced by factors related to their know-how or their experience [8], by pressure, as well as feared clinical complication and medical error [10], and by the unavailability of microbiological data [11]. For nurses, the lack of training and information [7, 12–14], missing recommendation guidelines, work overload, time constraints, and rejection by physicians are significant obstacles to the proper use of antibiotics [7, 14]. Finally, a lack of trust [15], a lack of resources allocated to pharmacies, a lack of awareness about the pharmacist's role, and a missing holistic vision around patient care [15, 16] prevent pharmacists from expressing their opinion on a prescribed antibiotic and its dosage. Therefore, it is essential to consider these different factors to bring about a sustainable change in practices favoring proper antibiotic use. Comparing professions and demonstrating their differences would enable the development of interventions adapted to the needs of each of them.

Given this fact, we surveyed the perceptions and attitudes of physicians, pharmacists, and nurses of a Belgian teaching hospital towards policies for the proper use of antibiotics. Therefore, this study aimed to identify barriers to the proper use of antibiotics by healthcare professionals and to help the hospital's Antimicrobial Stewardship (AMS) develop suitable actions for the staff.

## Methods

### COM-B model

Training is the tool most often used to change behavior, as it is associated with a positive and sustainable evolution of practices [17]. However, cultural, psychological, social, and institutional factors should be analyzed as they are significant levers for change [2]. A model capable of analyzing these levers is the « COM-B» model, derived from the “Behavior change wheel” (Additional file 1: Figure S1). It relies on three components: capability, opportunity, and motivation. To trigger behavior change, the three components have to interact [18, 19]. This approach identifies the different sources of behavior; targeting them through interventions could induce behavioral changes in health professionals in favor of careful antibiotic use. The choice of this model is justified by our will to use, as our reference framework for this work, the 2019 ECDC survey on attitudes, perceptions, and practices of health professionals toward antibiotic use and resistance [18]. Authors have thus analyzed health professionals’ perceptions as capabilities, access to resources as opportunities, and attitudes as motivations.

### Participants

This survey was conducted by the Antimicrobial Stewardship (AMS) of a 983-bed teaching hospital in Brussels, Belgium between January 15, 2020, and ended on February 20, 2020. We have decided to include qualified pharmacists, physicians, and nurses in the departments listed in Table 1 for their role in prescribing, dispensing, and administering anti-infectives. Students and trainees, pharmacists and nurses were excluded.

**Table 1:** Summary of inclusion and exclusion criteria

Inclusion criteria	Exclusion criteria
Physicians, pharmacists, and nurses working in the hospital	Not working in hospital
Physicians working in surgical, neuropsy, cardiovascular departments, internal, acute and dental medicine Nurses working in in surgical, neuropsy, cardiovascular departments, internal, acute medicine and mother child unit and mobile team Pharmacists of hospital	Working in psychiatry, Laboratory, Imaging, and palliative care departments
Working as physicians, qualified nurses, and pharmacists	Working as an assistant nurse, physiotherapist, paramedic, or students

### Questionnaire

We used the questionnaire from the ECDC 2019 survey [18] adapted to the hospital context and more specifically to the three professions selected (physician, nurse, and pharmacist). After validation of the protocol by our institutional ethics committee (N°2019/06Nov/487) and by the hospital's top management, the questionnaire was distributed by the institutional survey unit (Additional file 1: Survey questionnaire). The survey was put together with NetSurvey and sent through SurveyManager. This system automatically generates an untraceable link, which allows for anonymous responses.

### Statistical analysis

First, collected data were reviewed with the Ethnos software to analyze the different COM-B model components. For statistical analyses, responses were grouped into three clear-cut answers: agree, disagree, and no opinion, given the limited number of respondents in specific categories when nuances were made, and responses were classified into five categories (disagree, strongly disagree, no opinion, agree and strongly agree). Thus, to check whether the respondent's profession influenced the answers to the questions dealing with the different items (capabilities, opportunities, and motivation), contingency analyses were carried out using Fisher’s exact test: it tested the null hypothesis of the independence of the response score (agree, disagree, no opinion) according to the respondent's profession. A *p* value lower than 0.05 was considered significant.

## Results

**Fig. 1:**
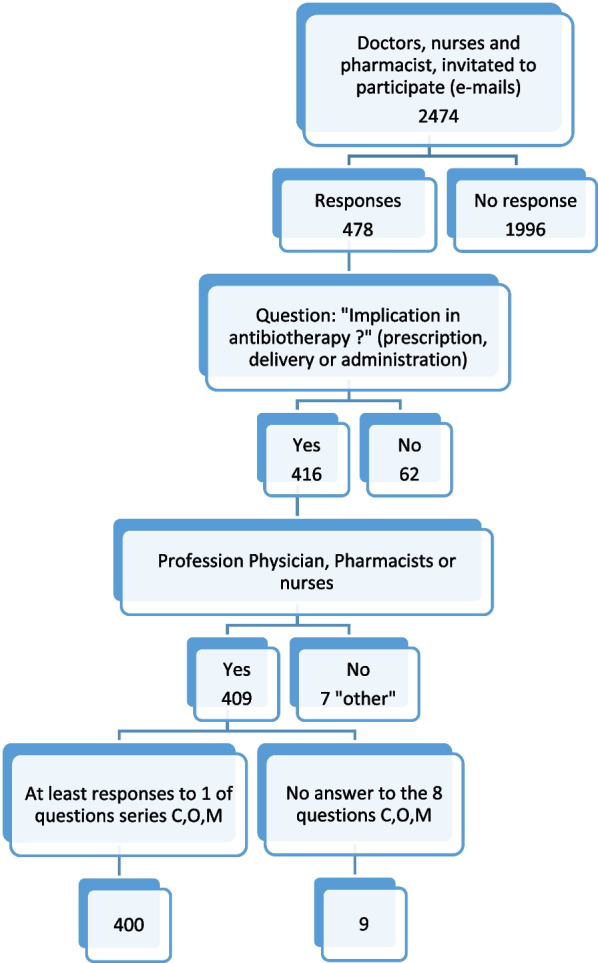
Participant selection process

A total of 2474 staff members who met the study inclusion criteria were invited to participate in the survey via email. Only 478 persons responded. For the 478 responses obtained, it was necessary to ensure all participants were involved in antibiotherapy. That is why a first question (Are you involved in diagnostic or prescribing activities, the clinical verification of prescriptions, the dispensing or administration of antibiotics, or an antibiotic therapy counseling activity, concerning patients or users/the public) was used to exclude 62 respondents. We exclude the professions of “others” (7). We also excluded professionals who had not answered at least one of the 8 COM-B questions (Fig. 1).

The sample (N = 400) comprised 127 physicians, 246 nurses, and 27 pharmacists. The participation rate per profession was 13.9% for physicians (127/912), 55.1% for pharmacists (27/49), and 16.3% for nurses (246/1513). Pharmacists (27) are overrepresented in our sample because they essentially answered the questionnaire (27/400), i.e., 6.8%, while they represent only 2% of the hospital staff. Physicians (127/400) are slightly underrepresented (31.8% of the sample versus 37.0% in the hospital), and nurses are represented in a good percentage (61.5% of the sample versus 61.1% in the hospital).

Survey results indicate that 64.3% of the sample (257/400), all professions combined, work full-time, and 41% (164/400) have over 15 years of service. Furthermore, 34.5% (138/400) of respondents are in the 25–35 age group. The distribution of respondents by the department doesn’t correspond precisely to the distribution of hospital staff in 2020. Additional file 1: Table S1 shows that for doctors, the departments most represented in relation to the hospital staff are internal medicine (28.4% vs. 25.7%), acute medicine (20.5% vs. 18.4%), surgery (19.7% vs. 17.5%) and pediatrics (12.6% vs. 9.6%). For nurses, the most represented departments are internal medicine (26.8% vs. 21.4%), acute medicine (19.9% vs. 12.7%), and mother–child care (17.5% vs. 16.1%). For doctors, dental medicine (2.4% vs. 9.3%), cardiovascular (4.7% vs. 7.4%), and necropsy (8.7% vs. 12.1%) are under-represented. For nurses, surgical (10.7% vs. 21.3%), cardiovascular (6.9% vs. 9.7%), neurosciences (6.9% vs. 9.7%) departments, and mobile teams (2.4% vs. 5.3%) are under-represented. 60.5% (242/400) of the sample do not contribute to antimicrobial stewardship programs and/or are not involved in the fight against antibiotic resistance.

### Capability analysis

Capability was assessed based on three questions (C1, C2, C3) concerning the respondent’s perceived feeling of knowledge and capability. We found that 97.8% (391/400) of all professions combined have a good perception of antibiotic resistance (C1). There was no statistical difference between the three professions (*p* = 0.733) (Table 2).

**Table 2:** Summary of results obtained for capability analysis depending on the profession

	Questions	Global hospital (%)	*p* value (Fischer)	Clear-cut opinion	No opinion
C1	I know what antibiotic resistance is	97.8	0.733	Pharmacist: 100.0% agree on Doctor: 98.4% agree on Nurse: 97.2% agree on	P: 0.0% D: 0.8% N: 0.4%
C2	I know which information to give to patients about the careful use of antibiotics and antibiotic resistance	87.5	0.0093	Pharmacist: 81.5% agree (14.8% disagree) Nurse: 84.2% agree (13.8% disagree)	P: 3.7% N: 2.0% D: 0.8%
				Doctor: 95.3% agree (3.9% disagree)	
C3	I have sufficient knowledge about the appropriate use of antibiotics for my current practice	77.3	< 0.0001	Pharmacist: 63.0% agree (37.0% disagree) Nurse: 71.5% agree (27.7% disagree)	P: 0.0% N: 0.8% D: 0.8%
				Doctor: 91.3% agree (7.9% disagree)	

For question C2 (Table 2), overall 87.5% (350/400) estimate that they know the information to give to the patients about the careful use of antibiotics and antibiotic resistance, with 95.3% for physicians, 81.5% for pharmacists, and 84.2% for nurses. The difference was statistically significant related to their profession (*p* = 0.0093). Furthermore, our results reveal that nurses and pharmacists are more likely to disagree with this statement than physicians. However, pharmacists are also more likely to have no opinion than physicians and nurses (Table 2).

Regarding knowledge about the appropriate use of antibiotics during their current practice (C3), 77.3% (309/400) state that they have sufficient knowledge, with 91.3%, 71.5%, and 63.0% for physicians, nurses, and pharmacists, respectively. There is also a statistically significant difference between respondents’ answers and their profession (*p* < 0.0001). Thus, pharmacists are more likely to disagree than other professions. No pharmacist indicated they had no opinion (Table 2).

### Opportunity analysis

Opportunities were assessed based on access to resources, information, and training (O1). It is observed that 72.2% (283/392) report having easy access to the guidelines they needed to manage infections. This overall score is 81.3% for physicians and drops to 69.2% and 67.9% for pharmacists and nurses. Only 39.5% (155/392) agree or strongly agree that they have easy access to the material (O2). In this context, only 59.2% (232/392) agree or strongly agree that they can advise patients or other healthcare professionals about the proper use of antibiotics*.* The results of the opportunity analysis are summarized in Table 3. Only the first question (O1) shows a statistical difference depending on the profession. Pharmacists’ answers are close to nurses', while the other two questions (O2 et O3) related to patient-centered opportunities do not show any significant differences by profession (*p* = 0.057 for O2 and *p* = 0.079 for O3).

**Table 3:** Summary of the results of opportunity analysis depending on the profession

	Questions	Global hospital (%)	*p* value (Fischer)	Clear-cut opinion	No opinion
O1	I have easy access to recommendations/guidelines I need to prescribe, check, prepare and administer antibiotics	72.2	0.00154	Pharmacist: 69.2% agree (30.8% disagree) Nurse: 67.9% agree (27.6% disagree)	P: 0.0% N:4.5% D: 0.0%
				Doctor: 81.3% agree (18.7% disagree)	
O2	I have easy access to the materials I need to advise on prudent antibiotic use, and antibiotic resistance	39.5	0.057	The three categories disagree: Pharmacist: 46.2% Doctor: 46.3% Nurse: 59.3%	P: 3.8% D: 4.9% N: 7.0%
O3	I have good opportunities to provide advice on prudent antibiotic use to individuals	59.2	0.079	Pharmacist: 57.7% agree (42.3% disagree) Nurse: 54.7% agree (40.8% disagree)	P: 0.0% N: 4.5% D: 4.9%
				Doctor: 68.3% agree (26.8% disagree)	

### Context analysis and identification of the problems encountered

Other questions provide a description of the context in which responses are obtained and an identification of specific causes of observed problems. Results reveal that 46.3% (185/400) and 34.0% (136/400) have prescribed, delivered, or administered an antibiotic, respectively, at least once a day and once a week over the past three months. Meanwhile, we also learn that 77.0% (308/400) have never distributed resources (flyers or brochures) about the careful use of antibiotics to patients or other health professionals and that 40.8% (163/400) state that they have rarely provided advice (at least once a month). Among the 24.3% of the participants who reported never having provided advice, we learn, based on a multiple-choice questionnaire with several possible answers, that the three most frequently listed reasons are: unavailability of resources (37.1%), lack of time (34.0%) and uncertainty about which advice to give (33.0%).

Furthermore, when asked about the most used resources in the management of infections, 50.5% of the participants first use an infectious disease specialist, 48.4% resort to recommendations and good practice guidelines, 47.6% rely on previous clinical experience, and 45.5% use informal exchanges between professionals.

In addition, the survey shows that 66.5% (266/400) of respondents have not received any information about the risks linked to inappropriate antibiotic therapy over the last 12 months. A multiple-choice questionnaire reveals that among those who received information (N = 109), 62.4% received it from colleagues, 53.2% from the workplace, and 45.0% and 44.0% from scientific publications and local guidelines, respectively. Training courses and conferences were mentioned in fifth place by 32.1% (N = 35/109). We also learned that, for 64.2% (70/109) of the respondents, this information changed their opinion on the useless or inappropriate prescription, administration, and delivery of antibiotics. Finally, 55.0% (60/109) reveal that this information has enabled them to change their practices.

When asked about the level at which the fight against antibiotic resistance is the most efficient, 48.5% (194/400) of participants rank prescriber education first, followed by patient education (33.2%) and education of all healthcare professionals (22.5%).

### Identification of solutions by healthcare workers

Furthermore, when asked about what they think are the three most efficient ways to raise awareness about the proper use of antibiotics and antibiotic resistance, 59.3% (237/400) want these topics to be included in continuing education, 53.3% (213/400) ask for recommendations and good practice guidelines about the management of infections, and finally, 32.5% (130/400) find it useful to be provided with posters and flyers on antibiotic awareness.

Finally, we adapted the last question of the questionnaire developed by the ECDC to our hospital environment by asking whether survey participants were aware of the existence of the AMS and its missions. It was found that 40.8% (163/400) report knowing about the AMS, and only 21.5% (86/400) report knowing its roles.

### Motivation analysis

The survey highlights that 92.8% (371/400) state that they know about the link between their practices and the emergence and spread of antibiotic resistance. However, only 65.0% (260/400) state that they have a role in managing antibiotic resistance. Once again, the results show that for the two motivation questions, there was a statistical difference dependent on the profession (for M1 (*p* = 0.0014) and M2 (*p* < 0.0001). The results reveal that pharmacists and physicians are more likely to agree, while nurses are often likely to disagree or have no opinion (Table 4).

**Table 4:** Summary of the results for motivation analysis depending on the profession

	Questions	Global hospital (%)	*p* value (Fischer)	Clear-cut opinion	No opinion
M1	I know there is a link between my prescription OR delivery OR administration of antibiotics and the emergence and spread of bacteria-resistant antibiotics	92.8	0.0014	Pharmacist: 96.3% agree (3.7% disagree)	P = 0.0%
				Doctor: 99.2% agree (0.0% disagree)	D = 0.8%
				Nurse: 89.0% agree (8.5% disagree)	N = 2.5%
M2	I have an important role to play in managing antibiotic resistance	65.0	< 0.0001	Pharmacist: 74.1% agree (25.9% disagree)	P: 0.0%
				Doctor: 85.0% agree (11.8% disagree)	D: 3.2%
				Nurse: 53.7% agree (40.6% disagree)	N: 5.7%

## Discussion

Our survey revealed that overall perceptions of antibiotic resistance are high. However, responses to several questions significantly depend on the profession for 5 out of 8 questions: for the two motivation questions, 2 out of 3 capabilities questions, and 1 out of 3 opportunity questions. Our study is in line with Barchitta et al. [20], reporting the Italian results of the ECDC survey [18], in which they also identify 3 clusters related to the profession and the activity. Indeed, cluster 1 consisted mainly of allied healthcare workers (HCW) with a strong majority of nurses (85.1%), and clusters 2 and 3 consisted of 99.8% of pharmacists and 99.7% of physicians, respectively.

Our results on respondents’ perceptions of antibiotic resistance (C1) are similar to the ECDC survey (97.8% versus 96%). There is no difference in knowledge (C3) about the proper use of antibiotics, where 77.3% of our participants agree against 80% for the overall ECDC sample and 78% for the whole Belgium sample. 87.5% of our participants seem to agree on knowing what information should be given to patients, compared to 86% for the ECDC sample and 84% observed for the whole of Belgium (Additional file 1: table S2). 

HCW in cluster 3 of Barchitta’s study had the highest antibiotic use and resistance knowledge. In our study, all three professions have a good knowledge of antibiotic use and resistance (C1 non-discriminating). Regarding capability (C2 and C3), it is also the physicians who are more confident in their knowledge as Cluster 3 (physicians) in the Italian study. Pharmacists and nurses estimate that they lack knowledge. They express interest and ask for antibiotic therapy to be integrated into their continuing education. Besides, analysis reveals that there are not enough organized training and information available at the hospital since 66.5% of respondents have not received any information over the last 12 months. Those who did (n = 109) obtained it from colleagues (62.4%) or the workplace (53.2%). Training courses and conferences only came fifth as sources of information for 32.1% of the sample (N = 109). However, not only are staff members willing to improve their knowledge, but it is observed that training courses/information influence their opinion (64.2% changed their opinion) and their practices (55.0% modified their practices). This demonstrates how the AMS can usefully train the three professional categories within the hospital. 

We identified two patient-oriented questions for the three opportunity questions and one related to professional activity. The three professions unanimously state that they lack patient-oriented opportunities: defective material or time to provide information and advice. The fact that responses to these two questions are not significantly different from one profession to another can be explained partly by the fact that they all work in the same environment. Access to structured and summarized patient information is lacking for everyone within the institution. For question O2, Barchitta et al. [20] also observe a similar response for the three professions. Still, the satisfaction rate (strongly agree and agree) is better with over 60%, whereas, in our study, it is only 39.5%. For the opportunities to advise individuals on prudent antibiotic use (O3), the percentage of participants who agreed or strongly agreed in the Italian study was higher in cluster 2 (90.2% pharmacist) and 3 (82.6% physicians) than in cluster 1 (64.9% nurse). In our study, the satisfaction rate (agree and strongly agree) is generally lower, with 68.3%, 57.7%, and 54.7% for physicians, pharmacists, and nurses, respectively. However, this difference between professions is not statistically significant (*p* = 0.079).

On the other hand, regarding opportunities related to daily practices (O1), responses to the question are significantly dependent on the profession with a statistical difference. Physicians agree (81.3%) that they have access to information/recommendations for their practice, whereas the situation is drastically different for pharmacists and nurses, who disagree (69.2% and 67.9% agree, respectively). In the Italian survey, physicians and nurses agreed with 70.5% and 68.5%, respectively, while pharmacists agreed with only 48.5%.

The third identified cause for lack of opportunity is the lack of knowledge, which can be related to one’s profession and perceived role in the issue. Access to recommendations is broadly comparable to ECDC results. The professionals at our hospital do not seem to have enough materials for managing infections, contrary to the results reported by the ECDC and for the entire Belgium sample. Furthermore, only 59.2% of our respondents say they have the opportunity to provide advice (O3), compared to 72.3% for the ECDC (Additional file 1: table S3). Nurses are the main drivers of the downward trend, while hospital nurses in the ECDC study say they have easy access to resources.

Furthermore, both studies found that health professionals do not always provide advice due to a lack of time and resources. On the other hand, the language barrier (12%) comes in the third position for the ECDC and only in the seventh position (7.2%) among our respondents; the third reason cited is the lack of certainty as to the advice be given (33/97). We learn that the three most used resources and the three sources of information the most requested by the respondents are the same in the two studies; however, according to degrees of different priorities.

Our hospital staff is just as open (or even more so) to change their opinion (64.2% against 58.3%) or practices (55.0% against 42.1%) as the European sample. According to the literature, nurses reported the same barriers to participating in antibiotic stewardship programs: time constraints, lack of knowledge of microbiology, and lack of understanding of antibiotics. The repression of doctors only appears with us in the fourth position, unlike the study by S. Abbas (20), where it appeared in the top 3.

For motivation issues, we found that the two motivation questions are the two most discriminating items between professions with a statistical difference (*p* = 0.0014 for M1 and *p* < 0.0001 for M2). It is observed that physicians (85.0%) and pharmacists (74.1%) are aware of the role they can play, while it is less the case for nurses (53.7%). The AMS must be able to demonstrate to nurses the role they can play through examples in their professional practice. For the Italian results of Barchitta et al. [20], the proportion of respondents who recognized their role in helping control antibiotic resistance (M2) was higher in clusters 3 (78.8% physicians) and 2 (78.6% pharmacists) than in cluster 1 (52.0% nurses). For question M1, the three professions strongly agreed or agreed that there is a connection between their prescribing/dispensing/ administering of drugs and the emergence and spread of antibiotic-resistant bacteria: 98.4% for cluster 3, 97.8% for cluster 1, and 95.8% for cluster 2. In our study, we have a significant difference between doctors (99.2%) and pharmacists (96.3%), with nurses agreeing at 89.0% (*p* = 0.0014). When we compare the motivation of the ECDC sample with that of our sample, we observe that the two populations are similar in awareness of the link (M1) between professional practice and antibiotic resistance. However, our respondents seem more inclined to recognize their role in controlling antibiotic resistance.

Indeed, 65.0% agreed with this statement compared to 56% for the European sample. But only 53.7% of nurses agree, while 85.0% of doctors and 74.1% of pharmacists agree.

In their current practice, it is shown that staff in all professional categories use infectious diseases specialists (50.5%), written recommendations (48.4%), previous clinical experience (47.6%), and informal exchanges between professionals during staff and interdisciplinary meetings (45.5%). Hospital healthcare professionals prioritize raising awareness through continuing education on antibiotic therapy and resistance and by issuing recommendations, good practice guidelines, and flyers/brochures on antibiotic awareness for staff and patients.

### Limitations

The most critical limitation of the study was the low participation rate. Despite this low participation rate (16.2%), we can guarantee a 95% confidence level with a threshold of 333 participants. Being above this threshold with 400 participants, we can validate and interpret these results. The medical and nursing departments most represented in the sample are concerned with antibiotic therapy. However, given the low participation rate of specific medical and nursing departments, particular attention from the AMS team should be paid to doctors in the cardiovascular and dental departments, nurses in the cardiovascular surgical departments, and mobile units. Despite this limitation, our study reinforces the idea that training, information, and tools should be targeted by profession. An analysis by professional sub-category would have added value to this study, even if the means available to our AMS do not always allow us to propose such a degree of personalization.

## Conclusions

Our results reveal that responses to questions are significantly dependent on the profession for 5 out of 8 questions: for the two motivation questions, 2 out of 3 knowledge questions, and 1 out of 3 opportunity questions. Our participants had good capabilities about the proper use of antibiotics but needed access to structured, summarized patient information and enough materials necessary for managing infections. Information is useless if it is not accessible. Communication is, therefore, also essential. This survey highlights the usefulness of infectious diseases specialists and the AMS in hospitals and has to motivate them to play their roles in managing antibiotherapy and antibiotic resistance. While some topics are universal/cross-functional, others must be specifically tailored to each professional category. We share the same conclusion as Barchitta et al. [20] with the need to develop training and tools tailored to each professional type.

## Supplementary Information

**Additional file 1.** Supplementary Figure, Tables and Survey questionnaire.

## Data Availability

The database is protected by GDPR for professionals.

## Abbreviations

AMS: Antimicrobial Stewardship
BAPCOC: Belgian Antibiotic Policy Coordination Commission
COM-B: Capability, opportunity, motivation, behavior
ECDC: European Center for Disease Prevention and Control
HCW: Healthcare workers
D: Doctor
N: Nurse
P: Pharmacist
