# Supplementary material for: Perceptions, attitudes, and practices of a Belgian teaching hospital's physicians, pharmacists, and nurses regarding antibiotic use and resistance: survey towards targeted actions for Antimicrobial Stewardship
Source: Antimicrob Resist Infect Control. 2023 Mar 19;12:19. doi: 10.1186/s13756-023-01228-w (PMC10024823; doi:10.1186/s13756-023-01228-w)
Supplement: Supplementary file 1 — Additional file 1. Supplementary Figure, Tables and Survey questionnaire. [file 13756_2023_1228_MOESM1_ESM.docx]

Figure S1: Relation between capability, opportunity, motivation, and behavior according to the COM-B model [19]

*Table S1: Comparison between the distribution of respondents and hospital staff in 2020.*

| **Medical departments** | Repartition of **doctors** by « department » in the survey (2020) | Repartition of **doctors** in Hospital (2020) | **Nursing departments** | Repartition of **nurses** by department in the survey (2020) | Repartition of **nurses** in Hospital (2020) |
| --- | --- | --- | --- | --- | --- |
| **Internal medecine** | 28,4% | 25,7% | **Internal medecine** | 26.8% | 21,4% |
| **Acute medecine** | 20,5% | 18,4% | **Acute medicine** | 19,9% | 12,7% |
| **Surgical department** | 19,7% | 17,5% | **Surgical department** | 10.7% | 21.3% |
| **Paediatrics department** | 12,6% | 9,6% | **Mother and Child unit** | 17,5% | 16,1% |
| **Neuropsy** | 8,7% | 12,1% | **Neurosciences** | 6,9% | 9,7% |
| **Cardiovascular Department** | 4,7% | 7,4% | **Cardiovascular Department** | 6,9% | 13,5% |
| **Dental Medecine (with stomato and maxillofacial)** | 2,4% | 9,3% | **Mobile teams** | 2,4% | 5,3% |
| **Unknown*** | 3,1% |  | **Unknown*** | 9,3 % |  |

* Unknown» : depatment non identified by the respondants

Table S2: Comparison of our results about capability with ECDC survey and overall Belgian sample

|  | Questions | Hospital | Global ECDC | Belgium ECDC |
| --- | --- | --- | --- | --- |
| C1 | I know what antibiotic resistance is. | 97.8% | 96% | >96% |
| C2 | I know which information to give to patients about the careful use of antibiotics and antibiotic resistance. | 87.5% | 86% | 84% |
| C3 | I have sufficient knowledge about the appropriate use of antibiotics for my current practice. | 77.3% | 80% | 78% |

*Table S3: Comparison of our results about opportunity with ECDC survey and overall Belgian sample*

|  | Overall | | | Doctors | | Pharmacists | | Nurses | |
| --- | --- | --- | --- | --- | --- | --- | --- | --- | --- |
|  | Hospital (%) | ECDC (Hospital) (%) | ECDC Belgium (%) | Hospital (%) | ECDC (Hospital) (%) | Hospital (%) | ECDC (Hospital) (%) | Hospital (%) | ECDC (Hospital) (%) |
| O1 | 72.2 | 75.7 | 73 | 81.3 | 83.1 | 69.2 | 84.5 | 67.9 | 70.7 |
| O2 | 39.5 | 64.2 | 63 | 48.8 | 69.9 | 50.0 | 76.6 | 33.7 | 58.8 |
| O3 | 59.2 | 72.3 | 72 | 68.3 |  | 57.7 |  | 54.7 |  |

**Survey questionnaire**

| **Perceptions, attitudes, and practices of physicians, pharmacists, and nurses, of a Belgian teaching hospital regarding antibiotic use and resistance: survey towards targeted actions for Antimicrobial Stewardship.** |
| --- |

**General information (check the answer(s))**

1. Are you involved in diagnostic or prescribing activities, clinical verification of orders/prescriptions, dispensing or administering antibiotics, or antibiotic therapy counseling activity, involving patients or consumers/public? *
   - Yes
   - Not

| **→if not:**  **Thank you for your interest in this investigation. However, for the objectives of the study an activity of prescribing, dispensing or administering antibiotics is required**. |
| --- |

1. What is your **main occupation** (i.e. >50% of your time)

Note: The following question is about your profession *

- - Physician (including surgeon, anaesthetist and assistant)
  - Nurse/midwife
  - Pharmacist
  - Other

1. At what regime do you hold your position in St-Luc ?

- Full-time (100%)
- 50% or more
- Less than 50%

1. **How many years** have you been in your current profession? *

- < 5 years
- 5 – 10 years
- >10 - 15 years
- > 15 years

1. **In which department** do you work? (Question if physician)

- Cardiovascular Department
- Department of Internal Medicine and Related Services
- Department of Surgery and Related Services
- Department of Pediatrics
- Department of Neuropsychiatry (Ophthalmology, ENT, Neurology)
- Departments of Acute Medicine (Emergency, Anesthesia and Critical Care)
- Department of Dental Medicine and Stomatology
- Infection prevention and control
- Other

1. **In which field** do you work? (question if nurse)
   - Loco-neuro-sciences
   - Cardiovascular sector
   - Mobility sector
   - Mother-child care
   - Acute Medicine
   - Internal Medicine
   - Onco-hematology
   - Operating area
   - Visceral branch
   - Infection prevention and control
   - other
2. How old are you? *

- ≤ 25 years
- >25-35 years
- >35-45 years
- >45-55 years
- > 55 years

1. In your current role, do you contribute to programs promoting the proper use of antimicrobials or are you involved in the fight against antimicrobial resistance?
   - Yes
   - Not
   - I don't understand the question

| 1. **Antibiotic use and resistance** |
| --- |

**1.1 To what extent do you agree or disagree with the following statements?***

1= strongly disagree; 2= disagree; 3= I don't know; 4= somewhat agree; 5= agree; 6= strongly agree; S/A= no notice

|  | 1 2 3 4 5 6 S/A |
| --- | --- |
| I know what antibiotic resistance is. | ○ ○ ○ ○ ○ ○ ○ |
| I know there is a link between my prescription OR dispensing or administration of antibiotics and the emergence and spread of antibiotic-resistant bacteria. | ○ ○ ○ ○ ○ ○ ○ |
| I know which information to give to patients about careful use of antibiotics and antibiotic resistance. | ○ ○ ○ ○ ○ ○ ○ |
| I have enough knowledge about the appropriate use of antibiotics for my current practice. | ○ ○ ○ ○ ○ ○ ○ |
| I have an important role in controlling antibiotic resistance. | ○ ○ ○ ○ ○ ○ ○ |

**1.2 Please indicate whether you believe these statements to be true or false.***

|  | True False I don't know |
| --- | --- |
| Antibiotics are effective against viruses. | ○ ○ ○ |
| Excessive use of antibiotics makes them ineffective. | ○ ○ ○ |
| Antibiotics can cause side effects, such as diarrhea or allergy. | ○ ○ ○ |
| Everyone treated with antibiotics has an increased risk of antibiotic-resistant infection. | ○ ○ ○ |
| Antibiotic-resistant bacteria can spread from person to person. | ○ ○ ○ |
| Healthy people can carry antibiotic-resistant bacteria. | ○ ○ ○ |
| The use of antibiotics to stimulate the growth of farm animals can have an impact on the ecology of bacteria in our environment. | ○ ○ ○ |

**1.3 To what extent do you agree or disagree with the following statements?***

1= strongly disagree; 2= disagree; 3= I don't know; 4= somewhat agree; 5= agree; 6= strongly agree; S/A= no notice

|  | 1 2 3 4 5 6 S/A |
| --- | --- |
| I have easy access to the recommendations/guidelines I need to prescribe, check, prepare and administer antibiotics. | ○ ○ ○ ○ ○ ○ ○ |
| I have easy access to the materials I need to advise on the prudent use of antibiotics and antibiotic resistance. | ○ ○ ○ ○ ○ ○ ○ |
| I have good opportunities to provide advice on the prudent antibiotic use to individuals. | ○ ○ ○ ○ ○ ○ ○ |

**1.4 In the last week of your clinical practice only, please assess the frequency with which the statements apply to you. If a question does not apply, please select "N/A.".***

|  |
| --- |
| How often have you prescribed OR dispensed OR administered antibiotics in the past 3 months?   - At least once a day - At least once a week - Rarely - Never |
| How often in the past 3 months have you distributed resources (e.g. pamphlets or brochures) on the prudent use of antibiotics or the management of infections to individuals (patients or healthcare professionals)?   - At least once a day - At least once a week - Rarely - Never |
| How often have you given advice on the prudent use of antibiotics or the management of infections to individuals (patients or healthcare professionals) over the past 3 months?   - At least once a day - At least once a week - Rarely (less than 1x/month) - Never |

| **Filter: go to the next question unless answer = never then go to question 1.5** |
| --- |

**1.5 If you have not been able to provide advice or resources as many times as you have prescribed** OR **dispensed OR administered antibiotics, what are the reasons?**

Indicate **all** appropriate answers

- The patient does not need information.
- The patient is not interested in the information.
- I have no contact with the patient
- Lack of time
- Difficulty in making the patient understand the diagnosis.
- Language barriers
- I give my advice to health professionals (nurses, doctors, pharmacists)
- No resources available
- I wasn't sure what advice to give.
- I was able to provide advice or resources as needed
- Other

**1.6 In the management of infections, who or what do you use regularly?**

Specify a maximum of 3 values.

- Recommendations/good practice guides on the intranet / PACO
- Pharmaceutical Industry Documentation/Industry Medical Representations
- Previous clinical experience
- Continuing Education Program
- Use of infectious disease specialists (calls)
- Scientific journals, professional resources/publications
- Internet
- Online continuing education
- None of these proposals
- Other

| 1. **Available information on antibiotic use and resistance** |
| --- |

**2.1 In the past 12 months, have you received any information to avoid prescribing OR unnecessary administration OR dispensing of antibiotics?***

- Yes
- Not

| **filter: If yes, proceed to the following questions 2.2 to 2.4** |
| --- |

**2.2 How did you first obtain this information to avoid prescribing OR administering OR dispensing** antibiotics  **unnecessary?***

Indicate **all** appropriate answers.

- Colleague
- My workplace
- Media Announcements (TV/Radio)
- Social Media
- Scientific journals, professional resources/publications
- Recommendations/Good Practice Guides
- Training – conference
- Training – in the form of individual interviews
- Government policy
- Professional organization on which I belong (example: AFPHB, Association of Physicians,...)
- Audit andfeedback carried out in my hospital
- Other

**2.3 Did this information help change your opinion about avoiding prescribing OR administering OR dispensing antibiotics unnecessarily?***

- Yes
- Not
- I don't know.

**2.4 Based on the information you have received, have you changed your prescribing OR unnecessary antibiotic administration OR dispensing practices?**

- Yes
- Not
- I don't know.

| 1. **Questions on information campaigns and training** |
| --- |

- 1. **At what level do you think the fight against antibiotic resistance is most effective?**  *

**Several** answers are possible.

- Education du patient
- Education of prescribers (physicians)
- Education of all health professionals
- Regional/national level
- European/global level
- I don't know.

**3.2 In your opinion, what are the 3 best ways to raise awareness about the proper use of antibiotics and the problem of resistance?**

- Short messages on screens near elevators
- Information by email sent by professional categories according to content
- Posters or pamphlets on antibiotic awareness
- Information in the hospital's internal journals: BIC and other journals
- Recommendations/good practice guides on infection management
- Subjects to be integrated into the continuing education of health professionals at least once a year
- Conferences/events on combating antibiotic resistance
- Day of awareness campaign within the CUSL
- E-learning accessible par l’intranet, podi-campus,…
- Other
  1. **What topics would you like to receive more information on?***

*Maximum 3 responses*

- Mechanisms of antibiotic resistance
- Therapeutic monitoring of certain anti-infectives. "Monitoring plasma antibiotic concentrations, why and how?"
- How to administer intravenous antibiotics ? Intermittent, prolonged or continuous infusion. Concentration and speed of administration.
- How to administer antibiotics in case of nasogastric and jejunal tube?
- What advice should be given to patients depending on the oral antibiotic prescribed?
- Recommendations for the use of antibiotics depending on the infection (choice of molecule, dosage and duration)
- Links between human health, animal health and the environment.
- Managing patient isolations: why isolate, when and for how long?
- I don't need information
- Other (please specify)

| Free fields |
| --- |

**3.4 In the hospital, do you know:**

|  | Yes No I don't know |
| --- | --- |
| The existence of the Antibiotic Management Group (AMG) | ○ ○ ○ |
| The roles (activities) of the AGM | ○ ○ ○ |
